# The Imaging of Guard Cells of *thioglucosidase* (*tgg*) Mutants of Arabidopsis Further Links Plant Chemical Defence Systems with Physical Defence Barriers

**DOI:** 10.3390/cells10020227

**Published:** 2021-01-25

**Authors:** Ishita Ahuja, Ralph Kissen, Linh Hoang, Bjørnar Sporsheim, Kari K. Halle, Silje Aase Wolff, Samina Jam Nazeer Ahmad, Jam Nazeer Ahmad, Atle M. Bones

**Affiliations:** 1 Department of Biology, Norwegian University of Science and Technology (NTNU), 7491 Trondheim, Norway; ralph.kissen@ntnu.no; 2 Cellular and Molecular Imaging Core Facility (CMIC), Department of Clinical and Molecular Medicine, Norwegian University of Science and Technology (NTNU), 7491 Trondheim, Norway; linh.hoang@ntnu.no (L.H.); bjornar.sporsheim@ntnu.no (B.S.); 3 Central Administration, St Olavs Hospital, The University Hospital in Trondheim, 7030 Trondheim, Norway; 4 Department of Mathematical Sciences, Norwegian University of Science and Technology (NTNU), 7491 Trondheim, Norway; Kari.Krizak.Halle@stolav.no; 5 National Centre for STEM Recruitment, Faculty of Information Technology and Electrical Engineering, Norwegian University of Science and Technology (NTNU), 7491 Trondheim, Norway; silje.wolff@ntnu.no; 6 Plant Physiology and Molecular Biology Laboratory, Department of Botany, University of Agriculture, Faisalabad 38040, Pakistan; saminatmalik@yahoo.com (S.J.N.A.); jamnazire@yahoo.com (J.N.A.); 7 Integrated Genomics, Cellular, Developmental and Biotechnology Laboratory, Department of Entomology, University of Agriculture, Faisalabad 38040, Pakistan

**Keywords:** abscisic acid, cuticle, glucosinolate-myrosinase system, rosette leaves, microscopy, myrosin cells, myrosinase, plant defence, stomatal guard cells, vacuoles

## Abstract

The glucosinolate-myrosinase system is a well-known plant chemical defence system. Two functional myrosinase-encoding genes, THIOGLUCOSIDASE 1 (*TGG1*) and THIOGLUCOSIDASE 2 (*TGG2*), express in aerial tissues of Arabidopsis. *TGG1* expresses in guard cells (GCs) and is also a highly abundant protein in GCs. Recently, by studying wild type (WT), *tgg* single, and double mutants, we showed a novel association between the glucosinolate-myrosinase system defence system, and a physical barrier, the cuticle. In the current study, using imaging techniques, we further analysed stomata and ultrastructure of GCs of WT, *tgg1*, *tgg2* single, and *tgg1 tgg2* double mutants. The *tgg* mutants showed distinctive features of GCs. The GCs of *tgg1* and *tgg1 tgg2* mutants showed vacuoles that had less electron-dense granular material. Both *tgg* single mutants had bigger stomata complexes. The WT and *tgg* mutants also showed variations for cell wall, chloroplasts, and starch grains of GCs. Abscisic acid (ABA)-treated stomata showed that the stomatal aperture was reduced in *tgg1* single and *tgg1 tgg2* double mutants. The data provides a basis to perform comprehensive further studies to find physiological and molecular mechanisms associated with ultrastructure differences in *tgg* mutants. We speculate that the absence of myrosinase alters the endogenous chemical composition, hence affecting the physical structure of plants and the plants’ physical defence barriers.

## 1. Introduction

The glucosinolate-myrosinase system is a well-known plant defence strategy towards insect herbivores and pathogens [1,2,3,4,5,6]. After cells are damaged by insect or pathogen attack, this defence system releases myrosinase (thioglucosidase) (thioglucoside glucohydrolase EC 3.2.3.147) from myrosin cells, and produces toxic compounds through hydrolysis of glucosinolates, a group of plant secondary metabolites [5]. The above-ground parts of the model plant Arabidopsis contain two kinds of myrosin cells, the guard cells in stomata, and phloem myrosin idioblasts [4,7,8,9]. Phloem idioblasts differ in size and morphology from neighbouring cells [8,10,11]. Stomata are specialised epidermal structures that are comprised of two guard cells (GCs) around a pore [12,13,14]. The pair of GCs acts as a gate to regulate the gas exchange activity against loss of water vapour through stomatal pores [15,16,17]. In general, there are two types of GCs: kidney-shaped and dumbbell-shaped. In dicots, GCs are kidney-shaped, and usually lack subsidiary cells, but they may also have two or more such cells [12,18]. However, in monocots, mature stomatal patterning can be categorized into different types depending on the presence, development, and arrangement of lateral subsidiary cells [19].

The stomatal pores, regulated by GCs, are crucial not only for CO2 uptake and control of water loss, but they are also entry sites for bacterial pathogens and some fungi [14,17,20,21,22]. A recent review has discussed the hypothesis that the glucosinolate-myrosinase system possibly has originated in stomata [23].

In Arabidopsis, myrosin cells are neighbouring vascular tissues, phloem, and procambium in the aerial parts, and are distributed on the abaxial side of the leaf relative to the phloem [2,7,8]. Although the molecular mechanism causing the development of myrosin cells is mostly unknown, the basic helix-loop-helix (bHLH) transcription factor FAMA, whose transcript and protein are specifically expressed in the stomatal lineage and acts as a master regulator of the terminal differentiation of GCs, is necessary for myrosin cell differentiation [2,9,24]. This outcome provided significant evidence for a link between myrosin cell development and stomatal development [2].

Two functional myrosinase-encoding genes, THIOGLUCOSIDASE 1 (*TGG1*) and THIOGLUCOSIDASE 2 (*TGG2*) are expressed in aerial tissues of Arabidopsis [25,26]. *TGG1* is expressed in guard cells and phloem cells while *TGG2* is only expressed in phloem-associated cells [1,8,27,28].

Several studies from Brassicaceae have shown the role of myrosinases in stomata and abscisic acid (ABA) related stress responses. Overexpression of a homologous TGG1 gene from broccoli (*Brassica oleracea* var. *italica*), BoTGG1, in Arabidopsis enhanced resistance against a bacterial pathogen by accelerating stomatal closure and inhibiting stomata reopening. Besides, BoTGG1-overexpressing plants were more sensitive to ABA-and salicylic acid (SA)-induced stomatal closure [29]. *tgg1* mutant plants were unresponsive to ABA inhibition of stomatal opening [28]. Islam and Co-workers presented a model for the role of TGG1 and TGG2 in the ABA and methyl jasmonate (MeJA) signalling network in GCs [30]. TGG1 and TGG2 are considered to function downstream of ROS production and upstream of cytosolic Ca^2+^ elevation in ABA and MeJA signalling.

Recently, through extensive metabolite and structural analysis of leaves of Arabidopsis thioglucosidase (*tgg*) mutants, we found a novel association between the glucosinolate-myrosinase defence system, a chemical barrier, and the cuticle, as a physical barrier [31]. This achievement led us on to further investigations into the structural characteristics of GCs of wild type (WT) and *tgg* single and double mutants. In the current study, and through the use of imaging techniques, we have found that the WT and *tgg1*, *tgg2* single, and double mutants show different and characteristic features for cell wall, chloroplasts, starch grains, stomatal ledges, and vacuolation in GCs. We have presented how the stomata and GCs appear in WT, *tgg* single, and double mutants, and what kind of variations they show for ultrastructure features. Additionally, through ABA treatment, we found that the stomatal aperture was reduced in *tgg1* single and *tgg1 tgg2* double mutants.

## 2. Methods

### 2.1. Plant Material and Growth Conditions

Seeds of Arabidopsis WT (Col-0), and *tgg1*, *tgg2* single mutants, and *tgg1 tgg2* double mutant lines [27] were stratified for two days at 4 °C and then transferred onto a soil mixture (1:1:1 peat moss-enriched soil/vermiculite/perlite) in 60 mm pots. The plants were grown in a randomised order in a growth-room at 22 °C/18 °C, 40/70% relative humidity, a 16/8 h light/dark period, and at 80 µmol m^−2^ s^−1^ light intensity for 3–4 weeks. To proceed for light microscopy (LM), scanning electron microscopy (SEM) and transmission electron microscopy (TEM) analyses, young rosette leaves of the WT and *tgg* single and double mutants were sampled 6 h after lights were switched on.

### 2.2. Microscopy Analyses

For SEM and TEM analysis, the methods by [20] were followed as reported earlier [31]. For SEM analysis, two young rosette leaves from two plants of each of the WT and *tgg* single and double mutants (about 3–4 weeks old plants) were cut in small square pieces and immediately fixed with glutaraldehyde (2%) in Sørensen’s phosphate buffer (0.1 M, pH 7.2) overnight at room temperature. After washing in buffer, the samples were dehydrated through a graded ethanol series at room temperature following the method [20]. After drying in a critical point dryer (Polaron Ltd., Hertfordshire, England) with liquid CO_2_, the samples were mounted on aluminium stubs for SEM and then coated with a thin layer (~30 nm) of gold-palladium using (2.5 kV, 20 mA) in a sputter coater Polaron coating unit E5100(Polaron Ltd., Hertfordshire, England). The images from the abaxial side were taken with a scanning electron microscope JSM-6480 (JEOL, Tokyo, Japan) and analysed using Scandium, universal SEM imaging platform [31]. The number of open and closed stomata in each SEM image were counted using software IrfanView 4.37. The area of 0.10 mm^2^, for each of the scanning electron micrographs of WT, *tgg* single, and double mutants was measured using the measure tool of Olympus iTEM software, the TEM imaging platform. For LM and TEM analysis, small pieces of tissues from young rosette leaves of WT and *tgg* single and double mutants (8–10 plants per genotype), were fixed with glutaraldehyde (2.5%) and paraformaldehyde (2%) in Sørensen’s phosphate buffer (0.1 M, pH 7.2) overnight at room temperature. Semi-thin resin-embedded sections (1 µm) were cut with an ultramicrotome Leica EM UC6 (Leica Microsystems, Wetzlar and Mannheim, Germany) using glass knives and stained with 0.2% toluidine blue solution for orientation, determine areas of interest, further trimming of the embedded blocks for ultra-thin sectioning. The sections were covered with toluidine blue solution and heated over an open flame. The sections were stained adequately during heating (for about 60 s), then washed thoroughly in 70% ethanol, and then in distilled water. The sections were allowed to dry and mounted with cytoseal and coverslip. Toluidine blue-stained semi-thin sections were observed under LM in order to see the variations in staining levels of stomata guard cells of WT and *tgg* single and double mutants. The slides with semi-thin sections were examined, and micrographs of the magnified images were made using 40× and 100× objective lenses (using microscope immersion oil) with a research microscope equipped with a digital camera ProgRes Capture Pro 2.10.0.1 (Carl Zeiss; Zeiss Axiophot, JENOPTIK Optical Systems GmbH, Jena, Germany).

From the areas selected, ultrathin sections (thickness = 70 nm) were cut using a diamond knife and collected on formvar-coated copper slot grids. The grids were stained with 4% uranyl acetate in 50% ethanol for 25 min, and alkaline lead citrate (1% in 0.2 M NaOH) for 5 min and then transferred to a grid box. The grids were viewed with a transmission electron microscope JEM 1011 (JEOL, Tokyo, Japan), equipped with a digital camera Morada, operating at 80 kV. The images were taken and processed using Olympus iTEM, the TEM imaging platform. The area of stomata from the TEM images was measured using the measure tool of Olympus iTEM, the TEM imaging platform.

For confocal imaging, we used a Leica TCS SP5 system attached to a DMI 6000 CS inverted microscope, equipped with an HCX PL APO CS 63X/1.2 NA water objective (Leica Microsystems, Mannheim, Germany). The green fluorescent fusion proteins were excited by the 488-nm Argon laser line, and the fluorescence emission was detected using a PMT in the spectral range 495–560 nm with a pinhole corresponding to 1 Airy unit, at 12-bit depth. The yellow fluorescent fusion proteins were excited by the 514-nm Argon laser line, and the fluorescence emission was detected using a PMT in the spectral range 520–570 nm with a pinhole corresponding to 1 Airy unit, at 12-bit depth. More than 20 biological replicates were used to analyse the TGG1 expression and subcellular localization, and 2–4 biological replicates were used in the case of the organelle markers (actin, ER and vacuole).

### 2.3. ABA Application and Estimation of Stomatal Aperture

Seeds of Arabidopsis WT (Col-0), and *tgg* single and double mutant lines [27] were sown onto a soil mixture in 30 mm pots. The plants were grown in parallel in a randomised order in a closed growth-cabinet at 22 °C/18 °C, 40/70% relative humidity, a 16/8 h light/dark period, and at 80 µmol m^−2^ s^−1^ light intensity. An ABA stock solution (1 mM) was prepared from (+/−)-ABA (Sigma-Aldrich) dissolved in 1 mL methanol and then brought up to 1 L with de-ionized water following method [32]. The control (mock) solution was prepared similarly while omitting ABA. The rosette leaves of WT, *tgg* single and double mutants were sprayed with 100 µM ABA and mock solutions twice a day on fully expanded rosette leaves of 21-d old plants. Five or six fully expanded leaves were harvested from the control and ABA treated six plants after three hours of the second ABA and mock applications, and immediately used to take impressions of leaves by applying low viscosity impression material, vinyl polysiloxane (silicone impression material) and hardener/catalyst (Zhermack), following the protocol described by Scarpeci and co-workers [33].

The mixture was applied on the abaxial surface of the leaves using a spatula and allowed to harden for about 5 min at room temperature. After hardening, the peel was taken off along the direction of the main leaf vein. A thin layer of clear nail varnish was applied to take an impression of covering the silicone leaf imprints and left to dry for 1 h at room temperature. The silicon rubber impressions were kept on a glass slide with a positive impression (surface covered with varnish) facing down. The thin layers of nail varnish were transferred to the glass slides by gently pressing the silicone rubber onto it and were covered with thin coverslips. The imprints were observed, and micrographs of the magnified images were made using a 40× objective lens with a research microscope equipped with a digital camera ProgRes Capture Pro 2.10.0.1 (Carl Zeiss; Zeiss Axiophot, JENOPTIK Optical Systems GmbH, Jena, Germany). The stomatal pore length and width were measured using software ProgRes Capture Pro 2.10.0.1, and the stomatal aperture was determined by calculating the ratio of width to length [33,34]. The ABA experiments were repeated twice under identical conditions and with the leaves harvested at the same time.

## 3. Results

### 3.1. tgg Mutants Differ for Stomata and Area of Stomatal Complex

**Figure 1:**
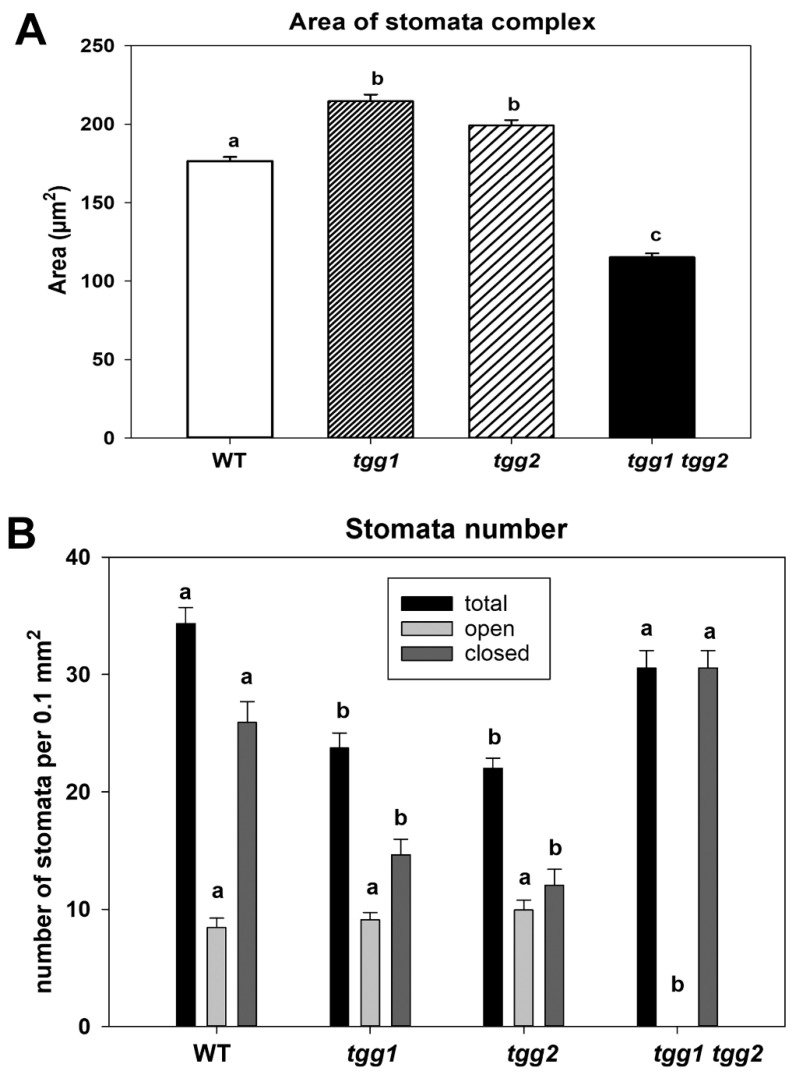
Area of stomata complex and number of stomata (total, open, and closed) assessed by scanning electron microscopy on rosette leaves of wild type (WT), *tgg1*, *tgg2*, and *tgg1 tgg2* double mutants of Arabidopsis [31]. (**A**). Area of stomata complexes. Bars represent the means ± SE (*n* = 60). Different letters above the bars indicate significant differences between WT, *tgg1*, *tgg2*, and *tgg1 tgg2* as determined for stomata complex by a Kruskal-Wallis test followed by a Dunn’s method for pairwise comparisons (*p* < 0.05). (**B**). Number of stomata (total, open and closed) per 0.1 mm^2^ of abaxial surface of rosette leaf. Bars represent the means ± SE. (WT: *n* = 23), (*tgg1*: *n* = 34), (*tgg2*: *n* = 31), and *tgg1 tgg2* (*n* = 29). Different letters (a, b and c) above the bars indicate significant differences between WT, *tgg1*, *tgg2*, and *tgg1 tgg2* as determined separately for each of the three categories (total, open, closed) by a Kruskal-Wallis test followed by a Dunn’s method for pairwise comparisons (*p* < 0.05).

Following the results from our previous publication [31], where we found significant variations for stomatal length among WT and *tgg* mutants, and for stomatal width between WT and *tgg1* single mutant, we further wanted to see how different genotypes differ for area of stomatal complex. The *tgg* single and double mutants differed significantly from WT for the area of stomata complex; Figure 1A [35]. Previously we observed that stomata of *tgg1* and *tgg2* single mutants were more open than those of the WT, and the stomata were closed in the *tgg1 tgg2* double mutant [31]. This led us to count the number of open and closed stomata for each of the genotypes. The total number of mature stomata and the number of closed stomata showed no significant differences for the WT and *tgg1 tgg2* but were lower for the *tgg* single mutants (Figure 1B). WT and *tgg* single mutants showed a significantly higher number of open stomata than *tgg1 tgg2*, as no open stomata were observed in the *tgg1 tgg2* double mutant.

### 3.2. The GCs Vacuoles of tgg1 Single and tgg1 tgg2 Double Mutants Lack Staining with Toluidine Blue

Toluidine blue has been used to stain leaf epidermal peels, stem epidermis, leaves, roots, myrosinase-containing myrosin cells (myrosin grains) [7,11,36,37,38,39], and to detect myrosinase [40,41]. In earlier studies, we showed that myrosin cells from semi-thin sections of transgenic *B. napus* seeds lacking myrosinase appeared empty or as empty holes after staining with toluidine blue [37,42]. On the other hand, the myrosin cells from the WT got densely stained after toluidine blue staining. Therefore, we used toluidine blue staining to reveal differences in GCs of WT and *tgg* single and double mutants.

**Figure 2:**
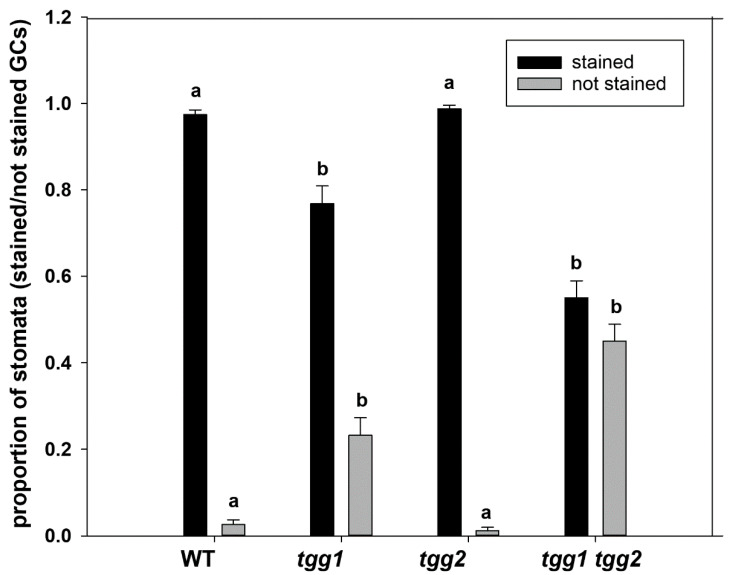
Proportion of stomata with guard cell (GC) stained or not stained (GCs lacking staining in vacuoles) by toluidine blue in light microscopy (LM) semi-thin sections (longitudinal) from rosette leaves of WT, *tgg* single and double mutants of Arabidopsis after staining with toluidine blue. Bars represent the means ± SE. The average of number of stomata with stained or not stained GCs per genotype were counted from semi-thin sections (WT: *n* = 14), (*tgg1*: *n* = 7), (*tgg2*: *n* = 12), and (*tgg1 tgg2*: *n* = 8). Different letters (a and b) above the bars indicate significant differences between WT, *tgg1*, *tgg2* and *tgg1 tgg2* as determined separately for each of the two categories (stained, not stained) by a Kruskal-Wallis test followed by a Dunn’s method for pairwise comparisons (*p* < 0.05).

**Figure 3:**
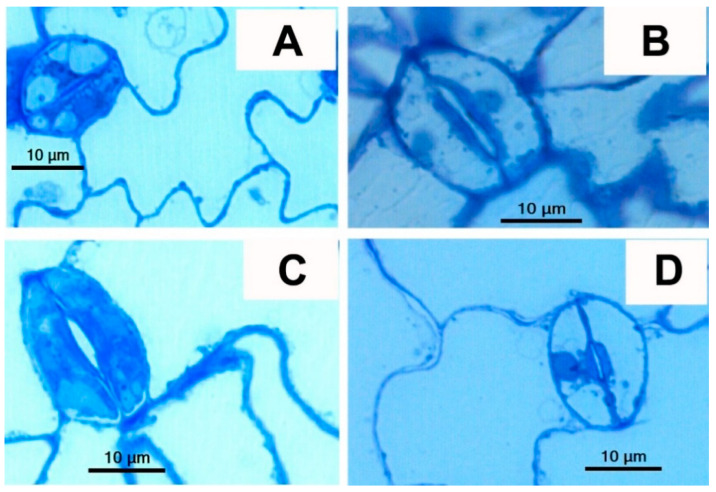
LM images of stomata and GCs from leaf segments of semi-thin sections (longitudinal) of rosette leaves of WT, *tgg* single and double mutants of *Arabidopsis* stained with toluidine blue. (**A**). WT: GC where vacuoles showed toluidine staining, (**B**). *tgg1* single mutant: GC where vacuoles lacked toluidine blue staining, (**C**). *tgg2* single mutant: GC where vacuoles showed toluidine staining, (**D**). *tgg1 tgg2* double mutant: GCs vacuoles showed no toluidine staining. (Scale bars = 10 μm).

The proportion of stomata with GCs stained by toluidine blue was significantly higher for the WT and *tgg2* single mutant than for the *tgg1* single and *tgg1 tgg2* double mutants (Figure 2, Figure S1). These differences in toluidine staining were mainly due to lack of staining in vacuoles of the *tgg1* single and *tgg1 tgg2* double mutants as compared to the WT and *tgg2* single mutant (Figure 2 and Figure 3, and Figure S1). Similarly, GCs (stomata complexes) from transverse sections of leaf segments of *tgg1* single and *tgg1 tgg2* double mutant showed a lack of staining in vacuoles when compared with the WT and *tgg2* single mutant (Figure S2).

### 3.3. ABA Treatment Affects Stomatal Aperture of tgg1 Single and tgg1 tgg2 Double Mutant

Based on the findings in previously published studies: that *TGG1* specifically expresses in GCs; that TGG1 overexpressing plants are more sensitive to ABA induced stomata closure; that *tgg1* mutant plants are non-responsive to ABA inhibition of stomatal opening; on the speculative role of TGG1 and TGG2 in ABA/JA signaling network [28,29,30]; and the differences among WT and *tgg* single and double mutants for area of stomatal complex and number of open and closed stomata (Figure 1), we wanted to extend our studies to see how the external application of ABA would affect stomata in these mutants.

**Figure 4:**
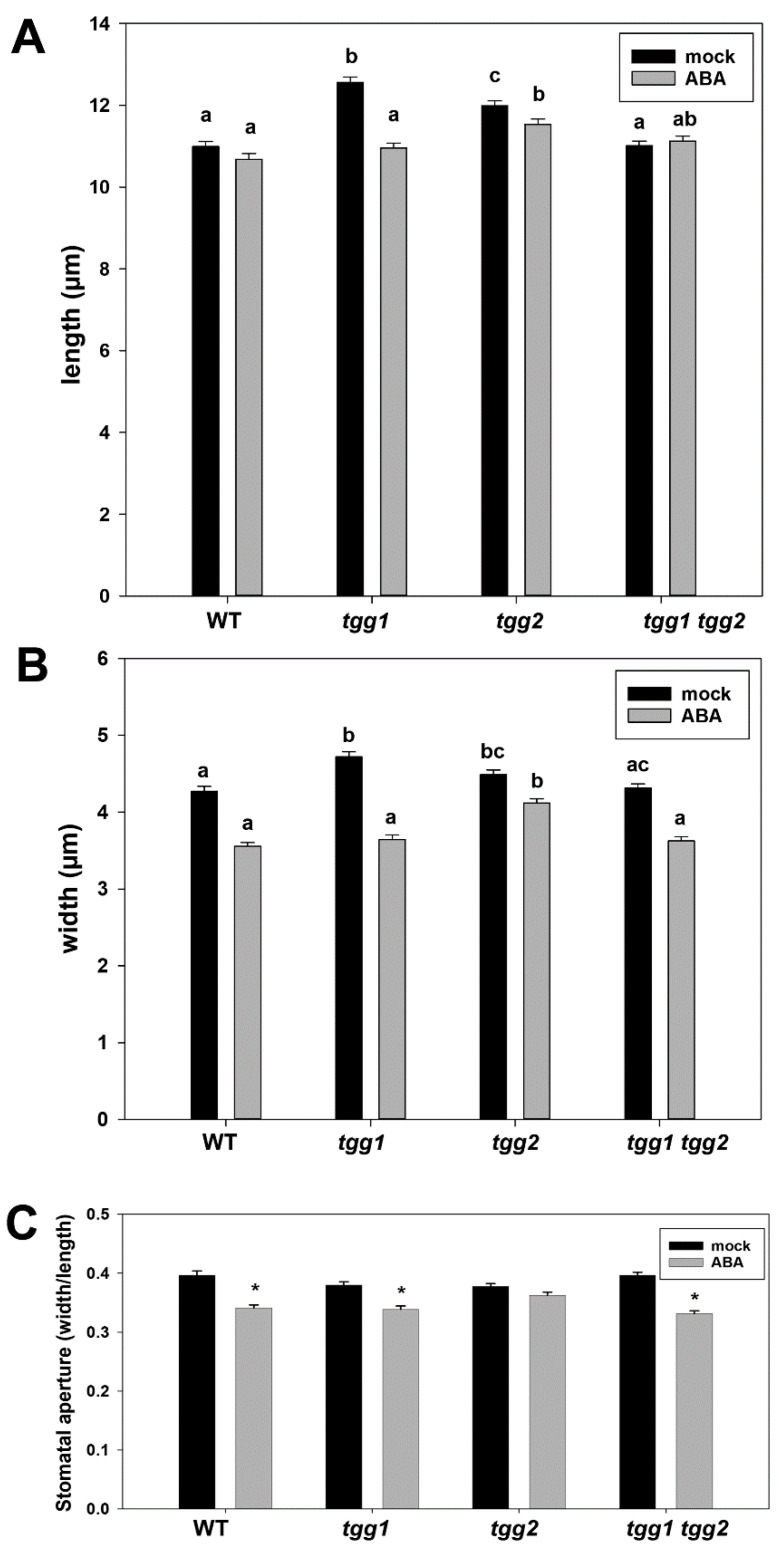
Effect of abscisic acid (ABA) (100 µM) on stomatal pore length, width, and stomatal aperture of WT and *tgg* single and double mutants. Length (**A**) and width (**B**) of the stomatal pore in WT, *tgg* single and double mutants after mock treatment or treatment with ABA. Different letters (a, b and c) above the bars indicate significant differences between WT, *tgg1*, *tgg2* and *tgg1 tgg2* for each treatment as determined by a Kruskal-Wallis test followed by a Tukey test for pairwise comparisons (*p* < 0.05). (**C**) Stomatal aperture in WT, *tgg* single and double mutants after mock treatment or treatment with ABA. Bars represent the means ± SE (*n* = 200). Asterisks above bars indicate a significant effect of the ABA treatment on stomatal aperture (* *p* < 0.05).

The effect of ABA on stomata in the different mutants was assessed by measuring the length and width of stomatal pores, and then calculating the stomatal aperture [33,34,43] (Figures S3 and S4). Under the control (mock) treatment, the stomatal pores of *tgg1* and *tgg2* single mutants were longer than those of the WT and the *tgg1 tgg2* double mutant (Figure 4A), and wider than those of the WT (Figure 4B). The *tgg2* single mutant differed significantly for length from the WT and *tgg1* single mutant after ABA treatment. We also observed that ABA treatment significantly reduced the stomatal aperture in the WT, *tgg1* single mutant and *tgg1 tgg2* double mutant (Figure 4C). Ten representative images of stomata from each of the mock and ABA treated *tgg1 tgg2* double mutant are presented in Figure S5 to show the differences in the width of the stomatal pore.

### 3.4. Ultrastructure Changes in Stomata and GCs of tgg Single and Double Mutants

**Figure 5:**
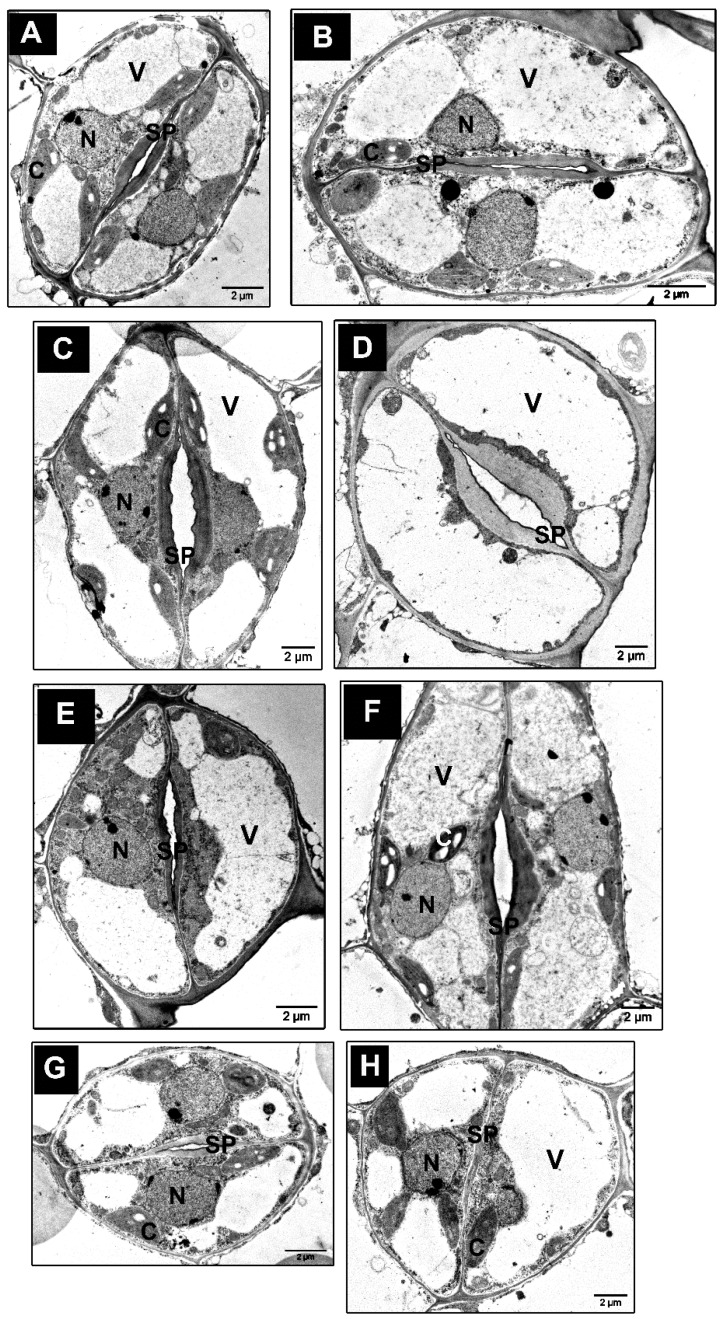
TEM of stomata and GCs from rosette leaves of WT, *tgg1*, *tgg2* single and *tgg1 tgg2* double mutants of *Arabidopsis*. (**A**,**B**). WT: GCs showing chloroplasts, open stomatal pore, nuclei, and small to big vacuoles in GCs. (**C**,**D**). *tgg1* single mutant: Showing vacuoles with less electron-dense material and open stomatal pores, (**E**,**F**). *tgg2* single mutant: (**E**). Stoma with slightly open stomatal pore, nucleus, with both small and big vacuoles, and chloroplasts with more prominent starch grains. (**F**). A bigger stoma with open stomatal pore; showing chloroplasts, nuclei, and big vacuoles in GCs with electron-dense granular material. (**G**,**H**). *tgg1 tgg2* double mutant: Small sized stomata showing vacuoles with less electron-dense material, chloroplasts, nuclei. C = chloroplast, M = mitochondria, N = nucleus, SP = stomatal pore, and V = vacuole. (Bars = 2 μm).

**Figure 6:**
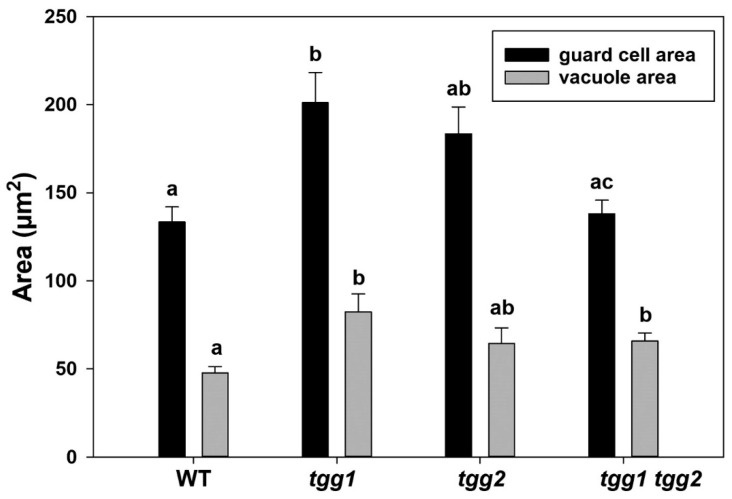
Area of GCs and vacuoles from transmission electron micrographs of rosette leaves of WT, *tgg1*, *tgg2* single, and *tgg1 tgg2* double mutants of *Arabidopsis*. Bars represent the means ± SE (WT: *n* = 12), (*tgg1: n* = 15), (*tgg2: n* = 15), and *tgg1 tgg2* (*n* = 18). Different letters (a, b and c) above the bars indicate significant differences in guard cell area and vacuole area between WT, *tgg1*, *tgg2*, and *tgg1 tgg2* as determined by a Kruskal-Wallis test followed by a Dunn’s method for pairwise comparisons (*p* < 0.05).

The occurrence of structural changes in GCs and stomata of WT and *tgg* mutants was further investigated by TEM. The GCs of the *tgg1* single mutant revealed bigger vacuoles with noticeably less electron-dense granular material (Figure 5C,D). Similarly, the vacuoles of *tgg1 tgg2* double mutant also showed less electron-dense granular material (Figure 5G,H). By contrast, the electron-dense granular material was visible in a higher amount in the vacuoles of GCs of the WT (Figure 5A,B), and *tgg2* single mutant (Figure 5E,F). The area of GCs was significantly greater for the *tgg1* single mutant than the WT, while the vacuolar areas of both *tgg1* single and *tgg1 tgg2* double mutant were significantly greater than the WT (Figure 6). The GCs of the *tgg1* single mutant (Figure 5C,D) were observed to be bigger than the WT (Figure 5A,B).

**Figure 7:**
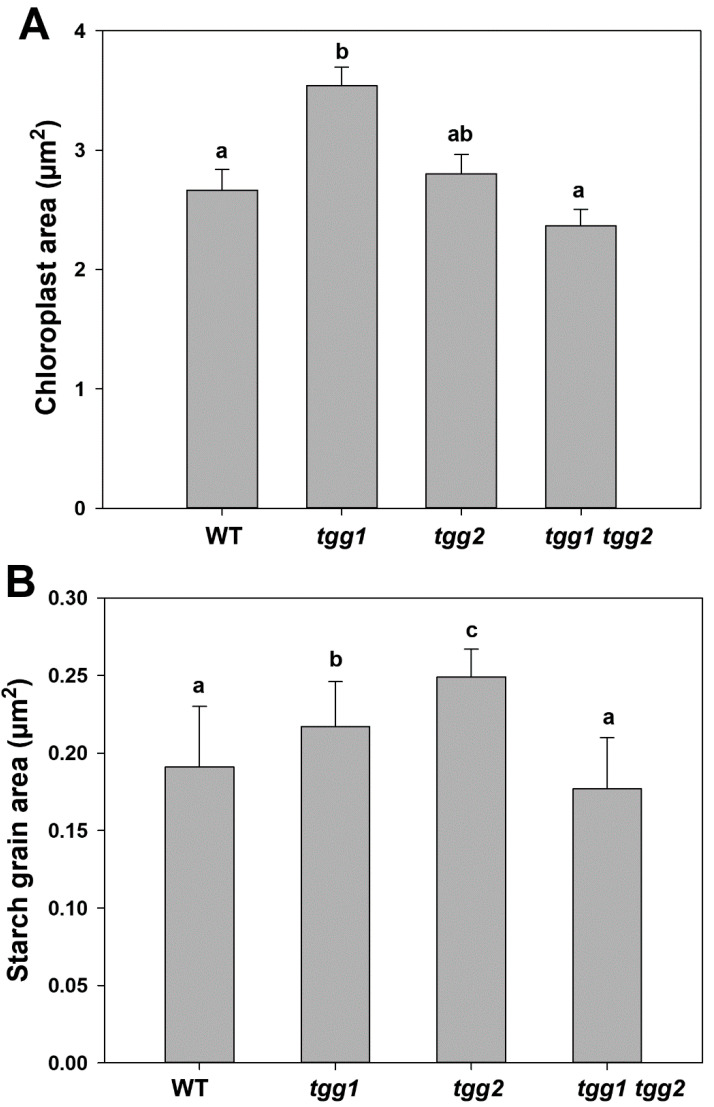
Chloroplast and starch grain area of guard cells from transmission electron micrographs of rosette leaves of WT, *tgg1*, *tgg2* single, and *tgg1 tgg2* double mutants of *Arabidopsis*. (**A**). Area of guard cell chloroplast in WT, *tgg* single and double mutants. Bars represent the means ± SE (WT: *n* = 49), (*tgg1: n* = 59), (*tgg2: n* = 35), and *tgg1 tgg2* (*n* = 41). Different letters (a and b) above the bars indicate significant differences in guard cell chloroplast area between WT, *tgg1*, *tgg2*, and *tgg1 tgg2* as determined by a Kruskal-Wallis test followed by a Dunn’s method for pairwise comparisons (*p* < 0.05). (**B**). Area of guard cell starch grains in WT, *tgg* single and double mutants. Bars represent the means ± SE (WT: *n* = 105), (*tgg1: n* = 159), (*tgg2: n* = 90), and *tgg1 tgg2* (*n* = 112). Different letters (a, b and c) above the bars indicate significant differences in guard cell starch grain area between WT, *tgg1*, *tgg2*, and *tgg1 tgg2* as determined by a Kruskal-Wallis test followed by a Dunn’s method for pairwise comparisons (*p* < 0.05).

**Figure 8:**
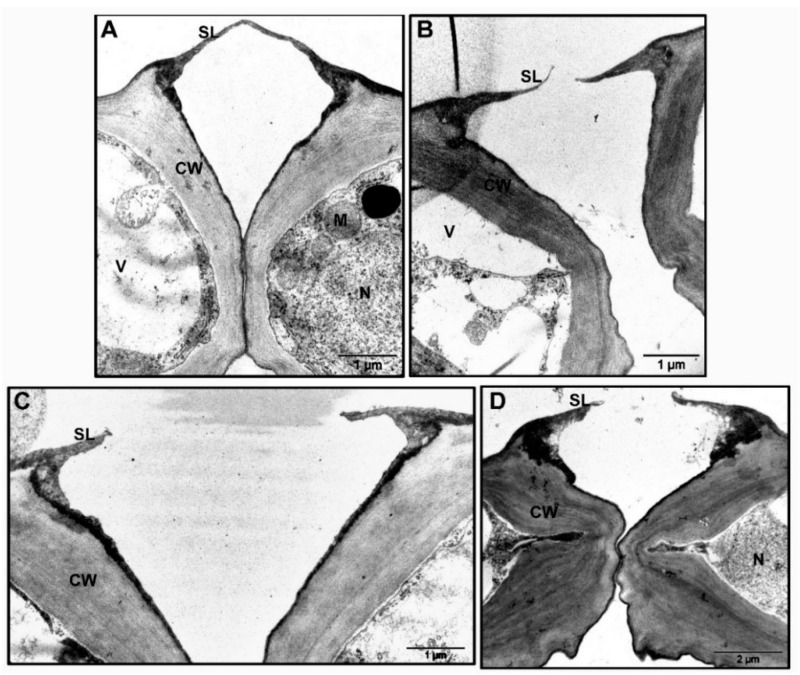
TEM of stomata complex and GCs (transdermal sections; adaxial side) from rosette leaves of WT, *tgg1*, *tgg2* single, and *tgg1 tgg2* double mutants of Arabidopsis revealed variations for outer stomatal ledge and cell wall. (**A**). WT: Stomata complex showing nucleus, mitochondria, and vacuole in either of the GC. Cell wall is differentially thickened with thickest near the stomatal pore, and thin between the GCs. The stomatal ledge is attached at the end of stomatal pore. (**B**). *tgg1* single mutant: Stomata complex showing vacuolated GC, with thick cell wall near the stomatal pore. GCs showing stomatal ledges at the end of stomatal pore. (**C**). *tgg2* single mutant: Stomata complex showing thick cell wall and reduced stomatal ledges at the end of stomatal pore. (**D**). *tgg1 tgg2* double mutant: Stomata complex showing very thick cell wall between the GCs and very thick stomatal ledges. CW = cell wall, M = mitochondria, N = nucleus, SL = stomatal ledge, and V = vacuole (Bars in **A**–**C** = 1 μm and in **D** =2 μm), (Magnification = 18,500× in **A**–**C**; and 11,000× in **D**).

**Figure 9:**
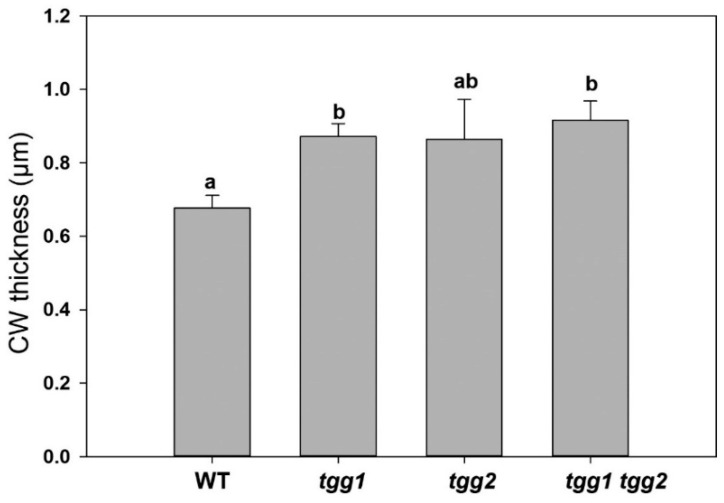
Cell wall (CW) thickness of guard cells as measured from transmission electron micrographs of rosette leaves of WT, *tgg1*, *tgg2* single and *tgg1 tgg2* double mutants of *Arabidopsis*. Error bars represent the means ± SE (WT: *n* = 17), (*tgg1: n* = 17), (*tgg2: n* = 8), and *tgg1 tgg2* (*n* = 20). Different letters (a and b) above the bars indicate significant differences in guard cell CW thickness between WT, *tgg1*, *tgg2* and *tgg1 tgg2* as determined by a Kruskal-Wallis test followed by a Dunn’s method for pairwise comparisons (*p* < 0.05).

We also observed chloroplasts with more prominent starch grains in the GCs of the *tgg2* single mutant compared to the WT, *tgg1* single, and the *tgg1 tgg2* double mutants (Figure 5). The area of the chloroplasts of *tgg1* single mutant was significantly greater compared to the WT and the *tgg1 tgg2* double mutant (Figure 7A). The area of starch grains in GCs of the *tgg1* and *tgg2* single mutants significantly differed from each other, and both showed a significantly greater area than the WT and *tgg1 tgg2* double mutant (Figure 7B). The GCs of the *tgg1* single mutant and the *tgg1 tgg2* double mutant showed thicker cell walls than the WT (Figure 8 and Figure 9).

Another interesting observation we made concerns the variations among WT, *tgg2* single, and *tgg1 tgg2* double mutants for stomatal ledges/cuticular ledges (lips) [44,45,46] (Figure 8 and Figures S6 and S7). Stomatal ledges appeared very thick in the GCs of the *tgg1 tgg2* double mutant (Figure 8D, Figures S6D and S7C,D), and shorter in the GCs of the *tgg2* single mutant compared to the WT (Figure 8C, Figures S6C and S7A,B).

During the maturation of GCs, the cell walls between the neighbouring GCs thicken and separate, and ultimately the surface facing the stomatal pore can be shaped, so that it leads to the formation of ledges that protrude from the upper edge of the ventral wall around each stomatal pore [38,46,47]. The outer cuticular ledge is considered to incline the orientation of stomatal pore to open and close, and to avert the entry of water droplets during the opening of stomatal pore [48,49]. It has been suggested that the ledges provide a recognition point for the pathogenic fungi that must find stomatal pores to get entry into their host plants [48,50]. Through a modelling approach, the outer ledges of some evergreen species were shown to prevent the wide opening of the stomatal pore and lifting above the leaf epidermis [51].

### 3.5. Arabidopsis Plants Expressing TGG1-GFP Shows GC Expression Pattern and Localization of TGG1 Fusion Protein in GC Vacuoles

**Figure 10:**
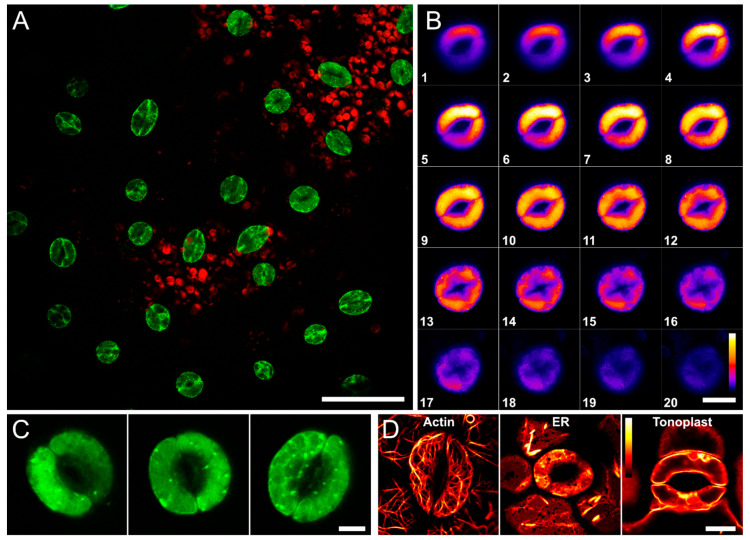
Confocal images of 17d old Arabidopsis rosette leaves (abaxial side) expressing TGG1-GFP, showing GC expression pattern (**A**) and subcellular localization (**B**,**C**), and 14-day old cotyledons marked with different fluorescent constructs labelling (**D**). (**A**). GCs (TGG1-GFP) shown in green and chloroplasts (autofluorescence) shown in red. Image is a maximum projection of ten optical slices in Z direction (Bar = 50 µm). (**B**). Twenty optical sections through a whole GC from top (1) to bottom (20), showing that the TGG1 fusion protein seem to localize within the GC tonoplasts/vacuoles (Bar = 10 µm) (**C**). High resolution, single sections from individual GC (Bar = 5 µm). (**D**). GCs expressing fluorescent markers for Actin (left), Endoplasmic reticulum (ER) (middle) and Tonoplast (right) (Bar = 10 µm). Actin was visualized by yellow fluorescent protein (YFP) fused to the actin-binding domain of mouse talin (mTalin) [53]. ER was visualized by YFP fused to a synthetic oligonucleotide encoding the ER retention signal HDEL (at C-terminus) and the signal peptide of AtWAK2 (*A. thaliana* wall-associated kinase 2; at the N-terminus) (ER-yk, CS16252) [53,54]. Tonoplast was visualized by YFP fused to the C-terminus of γ-TIP, an aquaporin of the vacuolar membrane (vac-yk; CS16258) [53,55]. Seeds of the transgenic *Arabidopsis thaliana* plants expressing the fluorescent markers for ER and tonoplast were obtained from Nottingham Arabidopsis Stock Centre (NASC).

Arabidopsis plants stably expressing a TGG1-GFP fusion protein under control of the TGG1 promoter showed the presence of TGG1 protein in GCs [8] (Figure 10A), with a subcellular localisation in vacuoles (Figure 10B,C). This corroborates earlier studies [7,28,52]. As we did not perform any colocalization, we visualised the localisation patterns of known organelles (Actin, ER, and vacuole) in GC for comparison purposes with TGG1-GFP (Figure 10D).

## 4. Discussion

To investigate the role of the glucosinolate-myrosinase plant chemical defence system in plant physical defence, we studied the stomata and GCs of WT, *tgg* single and double mutants by applying imaging-based approaches.

### 4.1. Scarcity of TEM Data on Stomata and GCs of Arabidopsis

A literature search showed that studies presenting TEM data on stomata or GCs from Arabidopsis are quite limited with the few existing examples representing work published by [12,20,39,45,46,49,56,57,58,59].

### 4.2. Bigger and Less Electron-Dense Vacuoles in GCs of tgg1 Single and tgg1 tgg2 Double Mutants Most Likely due to Lack of Myrosinase/TGG1

The bigger and less electron-dense vacuoles in GCs of *tgg1* single, and *tgg1 tgg2* double mutants compared to the WT and *tgg2* single mutant (Figure 5 and Figure 6), might be due to the lack of TGG1.

Moreover, in our previous studies with transgenic *Brassica napus* plants (where the idioblast myrosin cells were genetically ablated), using LM, TEM, and confocal microscopic approaches, we found the myrosin cells lacking myrosinase to appear more vacuolated [37,42]. Myrosin cells are structurally characterised by high protein content in the vacuole. Ultrastructural analysis of idioblast myrosin cells/guard-cell myrosin cells from a variety of plant families showed the characteristic features of moderately electron-dense homogeneous, and granular vacuolar material [6,7,10,11,60]. Consequently, we observed more electron-dense granular material in the WT, and *tgg2* single mutant, but not in the *tgg1* single, and *tgg1 tgg2* double mutants (Figure 5).

### 4.3. Stomatal Opening/Closing Possibly Affects Vacuolation and Stomatal Complexes in tgg Mutants

The area of stomatal complexes of *tgg* single mutants were significantly greater than the WT (Figure 1A). In our previous study, we showed the GC length to be considerably higher for *tgg* single mutants [31], which we also corroborate here (Figure 4A). The changes in the GC volume are considerable during the opening of stomata, which is mainly caused by a change in the plasma membrane and vacuolar surface area [16,17,61]. The enhanced opening of a stomatal pore in GCs of *tgg* single mutants also leads to the greater area of GCs in these mutants compared to the WT, and they appear bigger (Figure 5 and Figure 6). The GCs possess a high number of small vacuoles and several membrane structures in closed stomata [61,62,63]. However, during the stomatal opening, the small vacuoles and complex membrane systems fuse with each other or with bigger vacuoles to generate larger vacuoles. This can be seen in the transmission electron micrographs (TEM) of open stomata of the *tgg2* single mutant (Figure 5F), and open stomata of the *tgg1* single mutant (Figure 5C,D).

### 4.4. Cuticle, Transpiration, Stomatal Ledges, and Closed Stomata in tgg1 tgg2 Double Mutant

The cuticle controls transpiration through the stomatal pore via its role in forming the stomatal ledges, and a cuticular coating covering the mesophyll surfaces of the substomatal chamber [45,49,64]. These stomatal ledges, also referred to as cuticular projections, are a conserved feature of nearly all dicotyledonous GCs and are considered to prevent the penetration of water into the substomatal chambers [45,65].

The *tgg1 tgg2* double mutant showed very thick stomatal ledges, and the *tgg2* single mutant showed shorter/reduced stomatal ledges when compared to the WT (Figure 8 and Figure S6). In previous studies, the variations for stomatal ledges have been observed in stems of *wax2*, *cer9*, and *gpat4/gpat8* mutants of Arabidopsis [20,45,49]. We interpret that the thicker stomatal ledges in the *tgg1 tgg2* double mutant are either playing a role in controlling transpiration or preventing the penetration of water into the substomatal chambers. A recent study showed that plants lacking Fused Outer Cuticular Ledge (FOCL1) possessed malformed outer cuticular ledge, which forms a fused cuticular layer over the stomatal pores [38].

The SEM and TEM data from our studies showed stomata to be small and closed in the *tgg1 tgg2* double mutant [31]. The *tgg1 tgg2* double mutant with such a state of closed stomata is most likely showing the characteristics of a plant under drought stress (Figures S3 and S4) and thereby reducing water loss [31,66,67]. Myrosinase 2 (TGG2) was found to be one of the highly expressed proteins in *B*. *napus* in response to drought stress [68].

### 4.5. Chloroplasts, Starch Degradation in GCs, Glucosinolates, Myrosinases, and Responses to Blue Light

The area of GCs of chloroplasts of the *tgg1* single mutant was found to be significantly greater than the WT and *tgg1 tgg2* double mutant (Figure 7A). Additionally, the area of starch grains of *tgg1* and *tgg2* single mutants was also significantly greater than the WT and *tgg1 tgg2* double mutant (Figure 7B). Blue light triggers starch degradation in GCs [69], which has importance in plants’ adaptation to light through the control of stomatal aperture [70]. Earlier experiments also reported that the breakdown of starch in GCs is correlated with illumination of blue light [71], as referred by [57]. Further, the studies conducted by Lascève and co-workers showed that starch metabolism is necessary for full stomatal response to blue light [57]. Illumination with blue light enhanced the myrosinase activity and expression in radish hypocotyls [72,73]. Based on the available literature studies and the analysis performed by applying different protein localization tools, some of the glucosinolate biosynthesis proteins are found to be localised in the chloroplast [74]. Overall, the localization of some of the glucosinolate biosynthesis proteins in the chloroplast and the enhancement of myrosinase after illumination with blue light, highlight the occurrence of some unknown mechanisms in GCs, which need to be studied further.

### 4.6. Glucosinolates, Myrosinases, and Stomata/GC-ABA Responses

Exogenous stimuli like light, drought stress, pathogens and temperature tightly regulate stomatal aperture. These stimuli are sensed and signalled to the GCs via endogenous signalling molecules including phytohormones, where ABA is among the major players in terms of stress-related stomatal closure [28,43,75]. The increased area of the stomatal complex, stomatal pore-length, and width (Figure 1A and Figure 4A,B), again highlights the role of TGG1 in stomata/GC-ABA responses. TGG1 is indicated as a positive regulator in ABA inhibition of stomatal opening, and therefore to have an unrecognised role to play in plant abiotic stress responses [28]. A speculative model of interactions between the glucosinolates, myrosinase (TGG1), and K^+^ channels in GC-ABA signalling was presented [28]. A very recent study has also presented a model for the ABA signaling pathway in Arabidopsis guard cells [76]. Another recent study has shown that among others, glucosinolates and fatty acids were metabolites that were identified in *B*. *napus* guard cells and were found to be ABA responsive [77]. Additionally, treatment with the glucosinolate sinigrin promoted stomata closure in Arabidopsis and *B*. *napus*, with an additive effect by ABA treatment. This is consistent with an earlier study that showed that sinigrin-derived allyl isothiocyanate induced stomatal closure in Arabidopsis [78].

## 5. Conclusions

Through the imaging of stomata and GCs of Arabidopsis WT, and *tgg1*, *tgg2* single, and *tgg1 tgg2* double mutants, we have shown the ultrastructural differences between them for GC size, vacuolation, cell wall, chloroplasts, and stomatal ledge. In the current study, besides highlighting the limited availability of TEM data on stomata and GCs in Arabidopsis, we also raise questions about the physiological/molecular mechanisms that may be involved in generating bigger and less- electron dense vacuoles in *tgg1* single mutant/*tgg1 tgg2* double mutant and the enhanced opening of stomatal pore in *tgg1* single mutant. The results constitute a basis for a set of future experiments that should be performed with *tgg* mutants in order to better understand how changes in the glucosinolate-myrosinase system, a chemical defence system, may lead to the observed effects on physical defence barriers such as the cuticle, stomata and cell wall. These would include drought stress experiments, studying ABA mediated stomatal movement or investigating blue light responses, through the application of imaging, physiological, molecular, and omics approaches.

## Data Availability

The data presented in this study are available in the article and supplementary materials.

## Supplementary Materials

The following are available online at https://www.mdpi.com/2073-4409/10/2/227/s1. Figure S1: LM of mature stomata and GCs from leaf segments of semi-thin sections (longitudinal) of rosette leaves of WT, tgg single and double mutants of Arabidopsis stained with toluidine blue. Figure S2: LM of stomata complex and GCs from semi-thin sections (transverse) of rosette leaves of WT, *tgg* single and double mutants of Arabidopsis stained with toluidine blue. Figure S3: LM visualization of stomata on silicon imprints made from abaxial side of rosette leaves of WT, *tgg* single and double mutants of Arabidopsis in response to mock treatment. Figure S4: LM visualization of stomata on silicon imprints made from abaxial side of rosette leaves of WT, *tgg* single and double mutants of Arabidopsis in response to ABA (100 µM) treatment. Figure S5: LM visualization of stomata on silicon imprints made from abaxial side of rosette leaves from mock and ABA treated (100 µM) *tgg1 tgg2* double mutant of Arabidopsis. Figure S6: TEM of stomata complex and GCs (transdermal sections; abaxial side) from rosette leaves of WT, *tgg1*, *tgg2* single and *tgg1 tgg2* double mutants of Arabidopsis showing variations for outer stomatal ledge. Figure S7: TEM of stomata complex and GCs (transdermal sections) from rosette leaves of *tgg2* single and *tgg1 tgg2* double mutants of Arabidopsis showing variations for outer stomatal ledge.
