# Supplementary material for: The Imaging of Guard Cells of thioglucosidase (tgg) Mutants of Arabidopsis Further Links Plant Chemical Defence Systems with Physical Defence Barriers
Source: Cells. 2021 Jan 25;10(2):227. doi: 10.3390/cells10020227 (PMC7911204; doi:10.3390/cells10020227)
Supplement: Supplementary file 1 [file cells-10-00227-s001.pdf]

Supplementary Materials

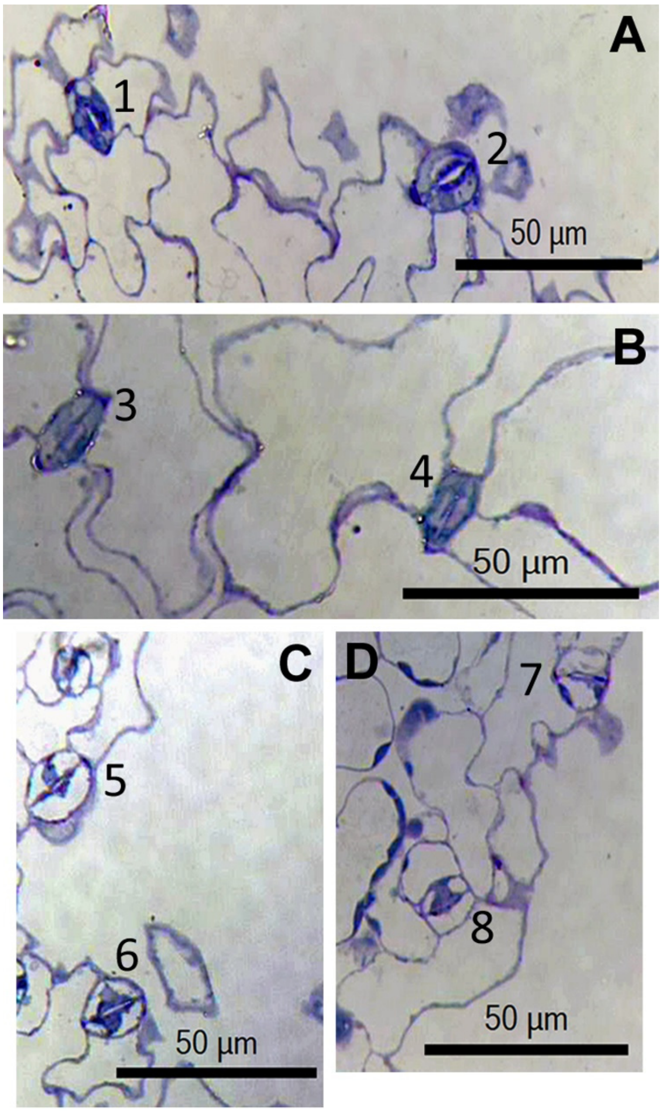

Supplementary Figure S1. LM of mature stomata and GCs from leaf segments of semi-thin sections (longitudinal) of rosette leaves of WT, *tgg* single and double mutants of *Arabidopsis* stained with toluidine blue.

A and **B.** Leaf segments of WT and *tgg2* single mutant showing variations in toluidine blue stained vacuolar material of stomata GCs. WT: 1 and 2; and *tgg2* single mutant: 3 and 4.

C and **D.** Leaf segments of *tgg1* single and *tgg1 tgg2* double mutant showing stomata GCs where the vacuoles appeared empty due to lack of staining of vacuolar material. *tgg1*: 5 and 6 and *tgg1 tgg2*: 7 and 8. (Scale bars = 50 µm).

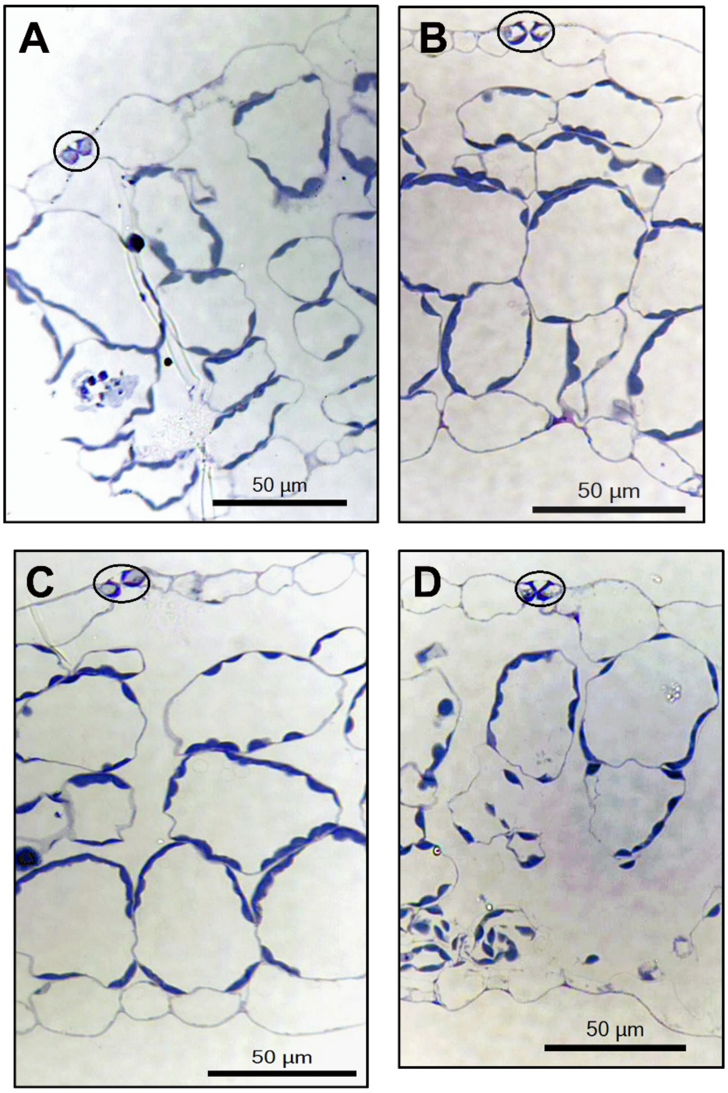

Supplementary Figure S2. LM of stomata complex and GCs from semi-thin sections (transverse) of rosette leaves of WT, *tgg* single and double mutants of *Arabidopsis* stained with toluidine blue. **A.** and **C.** WT and *tgg2* double mutant: Leaf segments showing toluidine blue stained stomata complexes and GCs (as shown in circles). **B.** and **D.** *tgg1* single and *tgg1 tgg2* double mutants: Leaf segments showing lesser stained stomata complexes, and lacking staining in vacuoles of GCs (as shown in circles). (Scale bars = 50 µm).

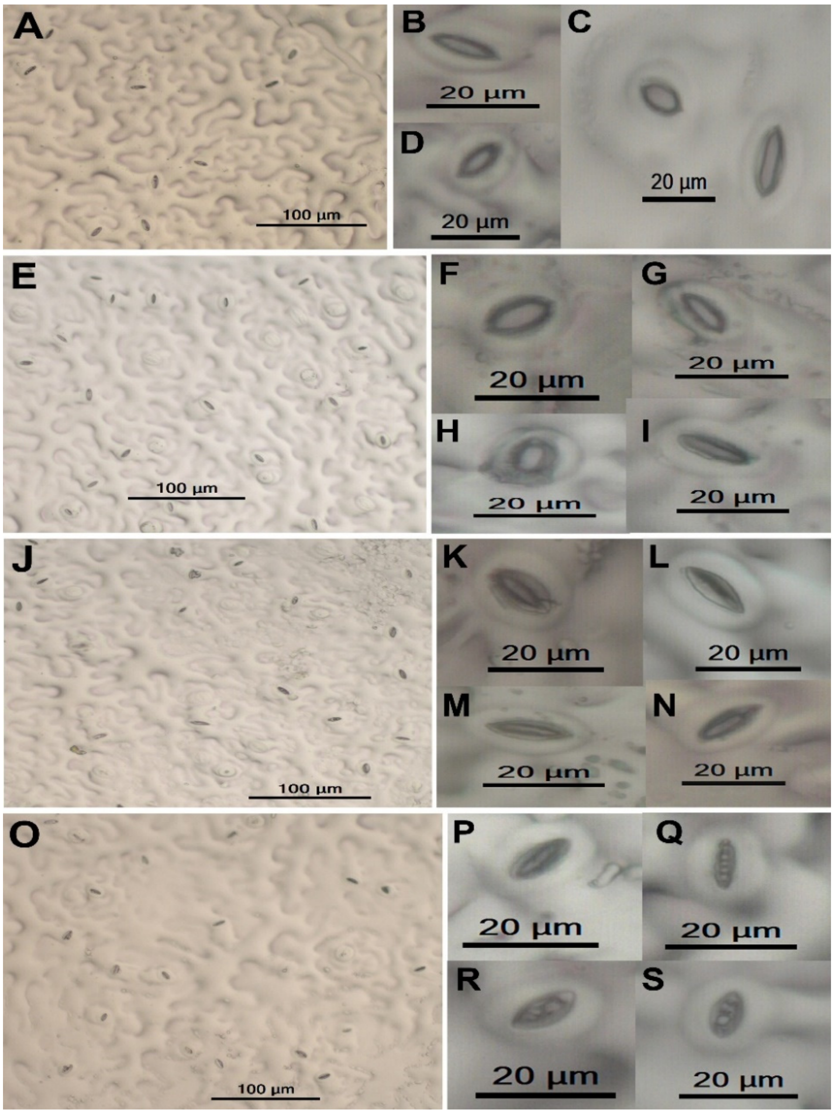

Supplementary Figure S3. LM visualization of stomata on silicon imprints made from abaxial side of rosette leaves of WT, *tgg* single and double mutants of *Arabidopsis* in response to mock treatment. **A - D.** Distribution of stomata as visualised on silicon imprints of leaves of WT (**A**), and images showing closer view of open stomata (**B - D**) of WT. **E - I.** Distribution of stomata as visualised on silicon imprints of leaves of *tgg1* single mutant (**E**), and images showing closer view of open stomata (**F - I**) of *tgg1* single mutant. **J - N.** Distribution of stomata as visualised on silicon imprints of leaves of *tgg2* single mutant (**J**), and images showing closer view of open stomata (**K - N**) of *tgg2* single mutant. **O - S.** Distribution of stomata as visualised on silicon imprints of leaves of *tgg1 tgg2* double mutant (**O**), and images showing closer view of closed stomata of *tgg1 tgg2* double mutant (**P - S**). (Scale bars for A, E, J and O = 100 μm) (Scale bars for B - D, F - I, K - N, and P - S = 20 μm).

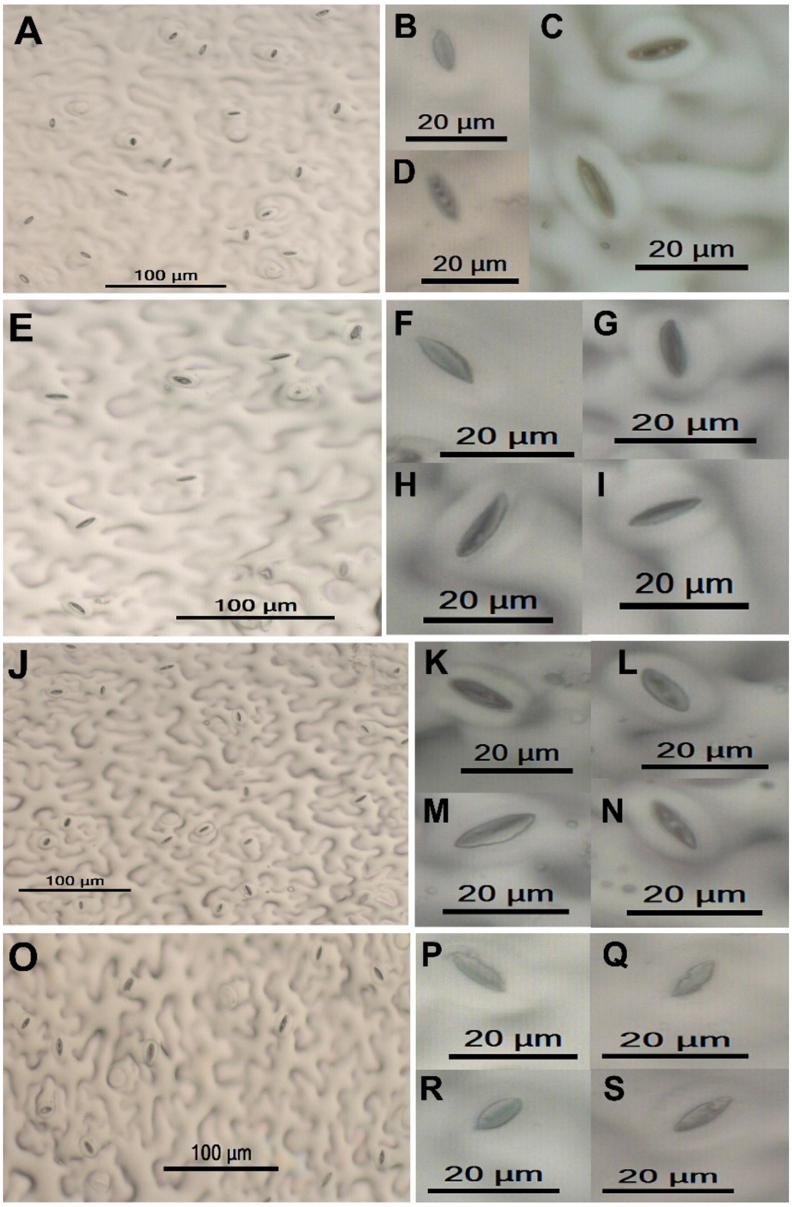

Supplementary Figure S4. LM visualization of stomata on silicon imprints made from abaxial side of rosette leaves of WT, *tgg* single and double mutants of *Arabidopsis* in response to ABA (100 µM) treatment. **A - D.** Distribution of stomata as visualized on silicon imprints of leaves of WT (**A**), and images showing closer view of closed stomata (**B - D**) of WT. **E - I.** Distribution of stomata as visualized on silicon imprints of leaves of *tgg1* single mutant (**E**), and images showing closer view of closed stomata (**F - I**) of *tgg1* single mutant. **J - N.** Distribution of stomata as visualized on silicon imprints of leaves of *tgg2* single mutant (**J**), and images showing closer view of closed stomata (**K - N**) of *tgg2* single mutant. **O - S.** Distribution of stomata as visualized on silicon imprints of leaves of *tgg1 tgg2* double mutant (**O**), and images showing closer view of closed stomata (**P - S**) of *tgg1 tgg2* double mutant.(Scale bars for (**A, E, J** and **O** = 100 μm) (Scale bars for B - D, F - I, K - N, and P - S = 20 μm).

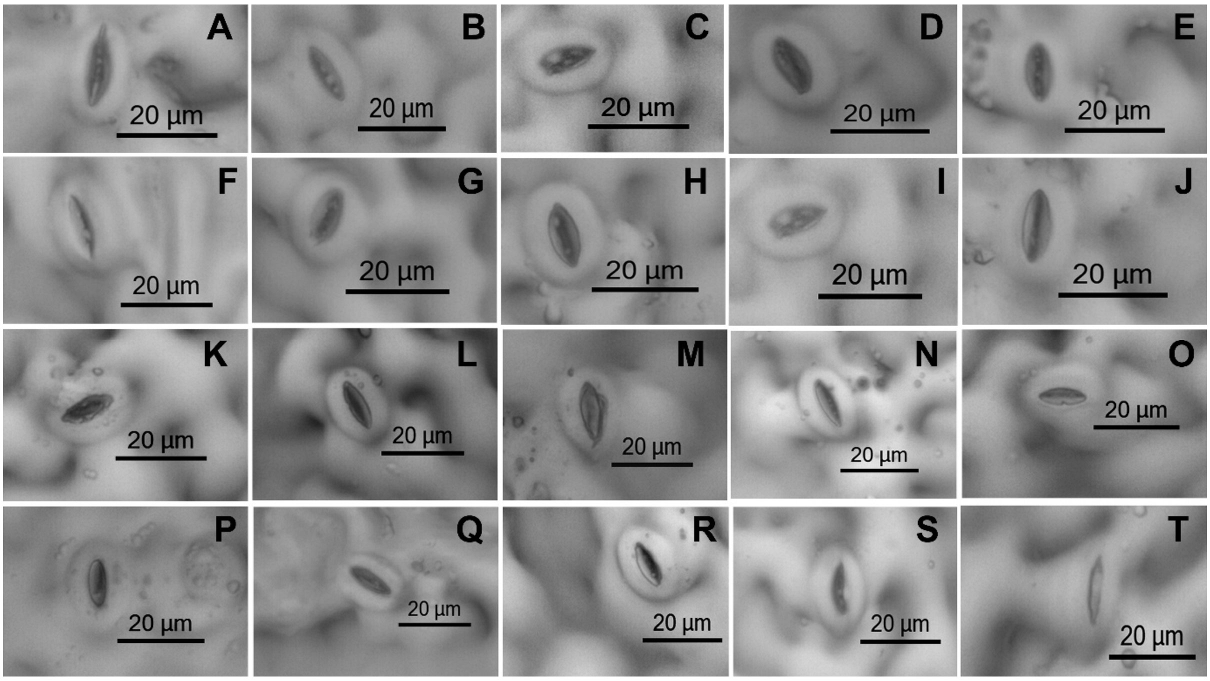

Supplementary Figure S5. LM visualization of stomata on silicon imprints made from abaxial side of rosette leaves from mock and ABA treated (100 µM) *tgg1 tgg2* double mutant of *Arabidopsis*. **A - J.** Stomata from leaves of mock treated plants. **K - T.** Stomata from leaves of ABA treated plants.

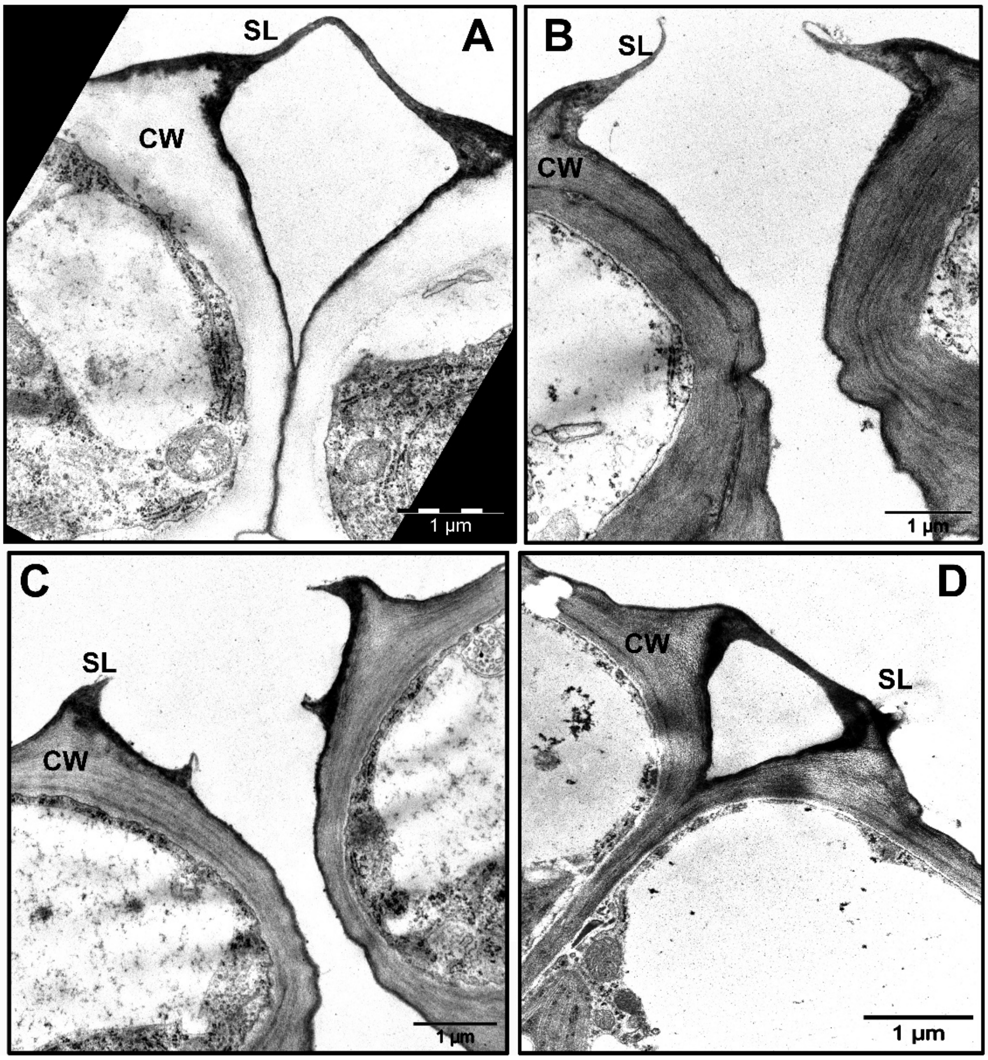

Supplementary Figure S6. TEM of stomata complex and GCs (transdermal sections; abaxial side) from rosette leaves of WT, *tgg1, tgg2* single and *tgg1 tgg2* double mutants of *Arabidopsis* showing variations for outer stomatal ledge. **A.** WT: The stomatal ledge is attached at the end of stomatal pore. **B.** *tgg1* single mutant**:** Stomata complex showing GCs with stomatal ledges at the end of stomatal pore. **C**. *tgg2* single mutant: Stomata complex showing GCs with reduced stomatal ledge. **D.** *tgg1 tgg2* double mutant: The stomatal ledge of stomata complex is thick and attached.

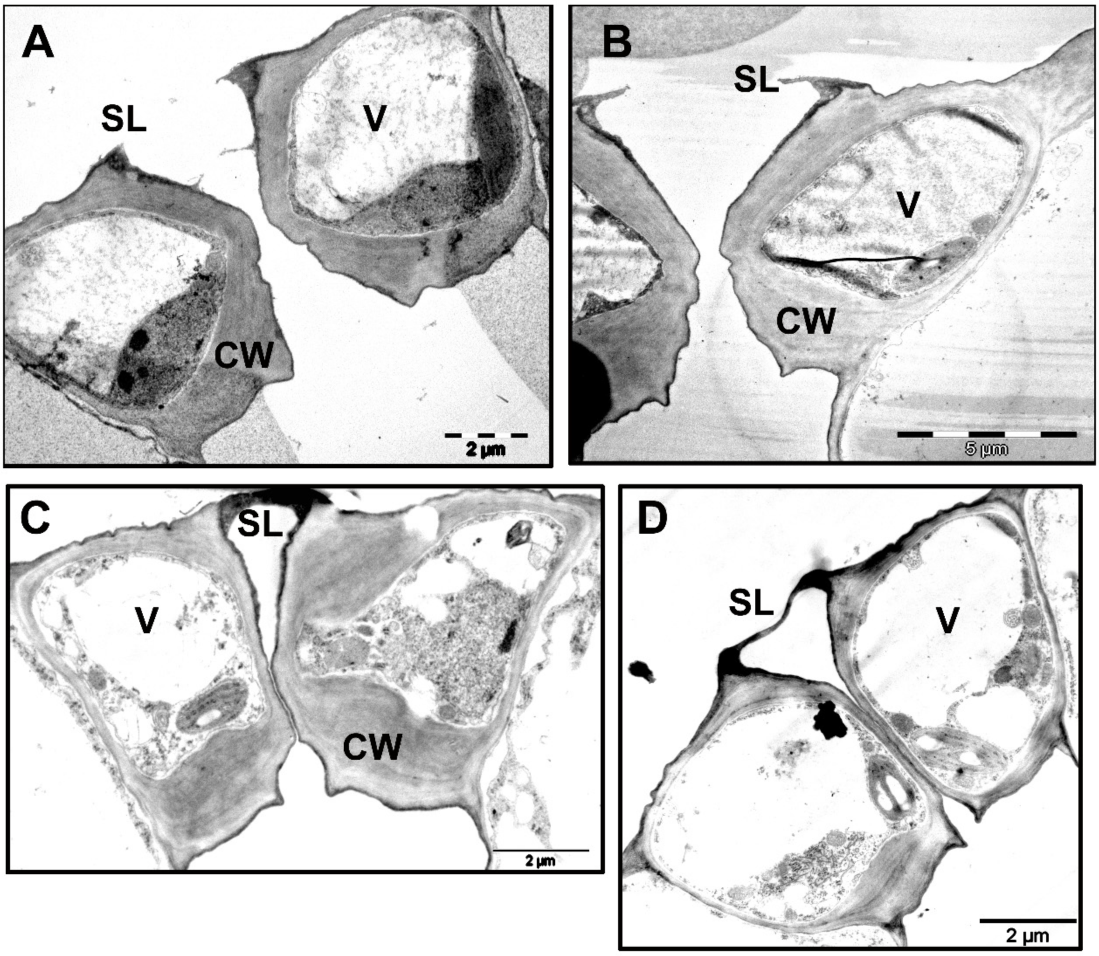

Supplementary Figure S7. TEM of stomata complex and GCs (transdermal sections) from rosette leaves of *tgg2* single and *tgg1 tgg2* double mutants of *Arabidopsis* showing variations for outer stomatal ledge. **A and B.** *tgg2* double mutant: Stomata complexes showing reduced stomatal ledges. **C** and **D.** *tgg1 tgg2* double mutant: The stomatal ledge of stomata complex is thick and attached.
